# Capacitive biophysical stimulation improves the healing of vertebral fragility fractures: a prospective multicentre randomized controlled trial

**DOI:** 10.1186/s10195-024-00758-2

**Published:** 2024-04-15

**Authors:** Andrea Piazzolla, Davide Bizzoca, Giovanni Barbanti-Brodano, Matteo Formica, Luca Pietrogrande, Umberto Tarantino, Stefania Setti, Biagio Moretti, Giuseppe Solarino

**Affiliations:** 1 UOSD Spine Surgery, AOU Consorziale Policlinico di Bari, Piazza Giulio Cesare 11, 70124 Bari, Italy; 2 Department of Spine Surgery, IRCCS Istituto Ortopedico Rizzoli, Bologna, Italy; 3 Department of Integrated Surgical and Diagnostic Sciences (DISC), University of Genova, Genoa, Italy; 4 Orthopedics and Traumatology Unit, Department of Health Sciences, San Paolo University Hospital, Azienda Socio-Sanitaria Territoriale Santi Paolo e Carlo, University of Milan Medical School, Milan, Italy; 5 Department of Orthopedics and Traumatology, Policlinico Tor Vergata (PTV) Foundation, Rome, Italy; 6 Clinical Biophysics, IGEA SpA, Via Parmenide, 10/A, 41012 Carpi, Italy; 7 Orthopaedic and Trauma Unit, Department DiBraiN, University of Bari “Aldo Moro”, Piazza Giulio Cesare 11, 70124 Bari, Italy; 8 Ospedale Policlinico San Martino, Genoa, Italy

**Keywords:** Acute vertebral fracture, Vertebral fragility fracture, Vertebral bone marrow edema, Osteoporosis, Osteopenia, Fragility fractures, Biophysical stimulation, Capacitive coupling electric fields (CCEF), MRI, Spine, Back pain, Ageing

## Abstract

**Background:**

Capacitively coupling electric fields (CCEF) is a method of non-invasive biophysical stimulation that enhances fracture repair and spinal fusion. This multicentre randomized controlled trial aimed to further examine the roles of CCEF in (1) the resolution of vertebral bone marrow oedema (VBME) using a follow-up MRI study and (2) pain relief, analgesic drug consumption and quality of life improvement in stimulated patients who were referred with acute vertebral fragility fractures (VFFs) compared to non-stimulated patients.

**Methods:**

Between September 2016 and December 2019, patients who were referred to the spine centres that participated in this multicentre randomized clinical study with acute VFFs of type OF1 or OF2 were included in the present study. All the VFFs were conservatively managed according to Good Clinical Practice. Moreover, the patients were randomized into two groups: the CCEF group received, as an adjunct to the clinical study protocol, biophysical stimulation with a CCEF device (Osteospine, IGEA) for 8 h per day for 60 days, whereas the control group was treated according to the clinical study protocol. At baseline (T0), the 30-day follow-up (T1), the 60-day follow-up (T2), and the 6-month follow-up (T3), each patient underwent clinical evaluation using the Visual Analogue Scale (VAS) for Pain and the Oswestry Disability Index (ODI). Analgesic therapy with paracetamol 1000 mg tablets for 7 days—or longer, depending on the pain intensity—was performed; patients were required to report their paracetamol consumption on a specific sheet between study day 8 to 180 days of follow-up. MRI studies of the thoracolumbar spine were performed at 0 (T0), 30 (T1) and 60 days of follow-up (T2) using a 1.5-T MRI system in all of the centres that took part in the study. For each VBME area examined via MRI, the vertebral body geometry (i.e. anterior wall height/posterior wall height and vertebral kyphosis) were assessed.

**Results:**

A total of 66 patients (male: 9, 13.63%; mean age: 73.15 years old) with 69 VFFs were included in the present study and randomized as follows: 33 patients were included in the control group and the remaining 33 patients were randomized into the CCEF group. In the CCEF group, good compliance with CCEF therapy was observed (adherence = 94%), and no adverse effects were recorded. In the stimulated patients, faster VBME resolution and significantly less vertebral body collapse during follow-up were observed compared to the control patients. Moreover, in the active group, faster pain reduction and improvement in the ODI mean score were observed. Stimulated patients also reported a significantly lower paracetamol consumption rate from the third follow-up after treatment until the 6-month follow-up. In terms of sex-related differences, in the CCEF group, VBME showed a faster resolution in male patients compared with females.

**Conclusion:**

Biophysical stimulation with CCEF, as an adjunct to traditional conservative treatment, is a useful tool to hasten the VBME resolution process and prevent vertebral body deformation. These MRI findings also correlate with faster back pain resolution and quality of life improvement. From the third follow-up after treatment until the 6-month follow-up, stimulated patients reported a significantly lower paracetamol consumption than control patients, even though back pain and quality of life showed no significant differences between the two groups.

**Level of evidence:**

II.

*Trial Registration* Register: ClinicalTrials.gov, number: NCT05803681.

## Introduction

Vertebral fragility fractures (VFFs) are the most frequent type of osteoporotic fractures, with a reported annual incidence of 700,000 in the USA and 620,000 in Europe [1]. In Italy, VFFs have an estimated annual prevalence of 61,000 and an overall incidence rate of 95.23 per 100,000 inhabitants [2]. Nonetheless, the prevalence of these fractures is significantly higher when considering patients aged 80 or older [2, 3].

These data, moreover, could be significantly underestimated, since approximately 60% of VFFs are clinically silent [4]. It is remarkable that asymptomatic VFFs can significantly impact the patient’s health by causing height loss, trunk deformity, impaired mobility, and an overall decreased quality of life [5].

Acute VFFs, which are identified by vertebral bone marrow oedema (VBME) on MRI, need orthopaedic management to prevent vertebral body collapse and alleviate back pain [6].

Unlike traumatic vertebral fractures, whose treatment is well standardized, the ideal management of VFFs is still debatable, and therapeutic strategies could differ between spine centres [3, 7–9].

Several percutaneous surgical techniques, including vertebroplasty, kyphoplasty, stentoplasty, and minimally invasive pedicle screw fixation, have been developed to improve the management of these fractures [3]. However, not all patients suffering from acute VFFs are suitable for surgical treatment because of their comorbidities, as highlighted by the AO Spine–DGOU Modified Score for Therapeutic Decision-Making in OF [7, 8].

Conservative management of VFFs consists of an initial period of bed with analgesia, followed by gradual mobilization—within the limit of pain—with a brace or corset, chosen depending on the fracture level [5]. Nonetheless, prolonged immobilization could impair elderly patients’ health by predisposing them to several comorbidities such as venous thrombosis, pulmonary embolism, pressure ulcers, and pulmonary and urinary tract infections [10].

Hence, in recent years, there has been a search for therapeutic protocols that could enhance the healing of VFFs, thus reducing bed-rest-related complications and improving the quality of life of osteoporotic patients. In this context, biophysical stimulation with capacitively coupling electric fields (CCEF) together with antiresorptive therapy, vitamin D supplementation and analgesic drugs could play a central role.

CCEF is a non-invasive type of biophysical stimulation used to enhance fracture repair and spinal fusion [5, 11]. In cases of osteoporotic vertebral fracture, positive effects of CCEF on chronic pain [1], postoperative pain, disability and quality of life after spinal fusion have been reported [12].

In a preliminary observational comparative study, Piazzolla et al. [5] showed that CCEF therapy provided significantly faster VBME resolution and back pain improvement in patients suffering from VFFs at the 3-month follow-up. However, to the authors’ knowledge, no multicenter, controlled, randomized trials have investigated the effectiveness of CCEF therapy on the healing of VFFs. Hence, a higher level of evidence is needed to integrate this therapeutic tool into daily clinical practice.

This multicentre randomized controlled trial aimed to further examine the roles of CCEF in (1) the resolution of VBME using an MRI study follow-up and (2) pain relief, analgesic drug consumption and quality of life improvement in stimulated patients who were referred with acute VFFs compared to non-stimulated patients. We hypothesized that biophysical stimulation with CCEF in combination with the standard conservative protocol for the treatment of VFFs could hasten VBME resolution. We also hypothesized that CCEF therapy, by hastening the fracture healing time, could prevent vertebral body deformation (defined based on the vertebral kyphosis angle [VK] and the ratio between the anterior wall height and the posterior wall height [AH/PH]) in patients treated with an initial period of bed rest followed by bracing.

We finally hypothesized that a faster VBME resolution is related to more rapid back-pain resolution (in terms of the Visual Analogue Scale for Pain [VAS] and the paracetamol consumption) and quality of life improvement (as measured by the Oswestry Low Back Disability Index—ODI).

## Material and methods

This study was designed as a prospective and randomized multicentre clinical trial with two groups: a study group (CCEF group) and a control group.

Patients who were referred to the spine centres that participated in this multicentre randomized controlled trial with acute VFFs of type OF1 or OF2 [8] between September 2016 and December 2019 were included in the present study. Ethical clearance was obtained from our centre’s clinical research ethics (study no. 4920, protocol no. 25,788/2016, March 30th, 2016, Comitato Etico Indipendente, AOU “Policlinico”), as per the 1964 Declaration of Helsinki, and all patients gave their informed consent before enrolment in the study. The study protocol has been registered on ClinicalTrials.gov (NCT05803681).

### Patient selection

Inclusion criteria were: male and female; age ≥ 60 years old; BMI ≤ 35 kg/cm^2^, since a higher BMI could affect the CCEF distribution; fracture site between T10 and L3, so that the same brace, the three-point hyperextension brace, was used; symptomatic acute VFF, with acute pain at the fracture level; low back pain onset within 20 days; and VBME > 60% according to MRI at baseline.

Exclusion criteria were: posterior wall/pedicle injury, since surgical treatment is recommended for such findings; previous vertebroplasty/kyphoplasty, which could impair the VBME quantification; a history of spine infection or tuberculosis, as we wished to exclude diseases that could overlap with the fragility fracture; a history of malignant tumours that could spread to the spine, as we wished to exclude diseases that could overlap with the fragility fracture; concomitant rheumatoid arthritis or spondyloarthritis, as we wished to exclude diseases that could overlap with the fragility fractures; scoliosis ≥ 40° according to Cobb, thoracolumbar kyphosis > 20° and/or thoracic kyphosis > 70°, as we wished to exclude some conditions that could cause chronic back pain; any contraindication to MRI, since MRI is required to detect VBME resolution; and the use of biomedical devices that could interfere with CCEF biophysical stimulation.

All the VFFs in both the active and control groups were conservatively managed according to Good Clinical Practice, i.e. all the patients received (1) bed rest for the first 20 days and subsequent mobilization with a three-point hyperextension brace; (2) antiresorptive therapy (a 75-mg risedronate tablet weekly); (3) supplemental calcium carbonate with 1000 mg of elemental calcium daily if needed based on the serum calcium concentration; and (4) vitamin D (≤ 500 IU daily) if the serum 25-hydroxyvitamin D concentration at the time of screening was below 16 ng/ml.

Analgesic therapy with paracetamol 1000 mg tablets for 7 days—or longer, depending on the pain intensity—was performed; patients were required to report their paracetamol consumption on a specific sheet.

### CCEF protocol

The recruited patients were randomized into two groups: the CCEF group and the control group. In the CCEF group, patients received, as an adjunct to the clinical study protocol, CCEF stimulation (Osteospine^®^, IGEA SpA, Carpi, Italy) for 8 h/day for 60 days, whereas in the control group, patients were treated according to the clinical study protocol.

Patients were randomly assigned to the CCEF group or control group using a web-based randomization program built on the following randomization criteria: BMI (19 ≤ BMI ≤ 25; 26 ≤ BMI ≤ 35), sex (male/female) and smoking status (yes/no). Data were collected and inserted into a clinical report form for each patient.

Capacitive biophysical stimulation was performed using the device Osteospine^®^ (IGEA SpA, Carpi, Italy), a medical device that weighs 140 g and provides a current density of up to 30 µA/cm^2^ in the region of interest. The device signal consists of a 12.5Hz burst with a duty cycle of 50%. The active part of the burst is a sinusoidal wave of 60 kHz ± 4% with an amplitude adjusted by a microprocessor according to the impedance of the body interposed between the electrodes.

The pad is made of highly conductive material covered with adhesive gel. Previous studies have shown good skin tolerability of the device [5, 11]. The devices used in the present study had built-in software to record the stimulation times.

**Fig. 1:**
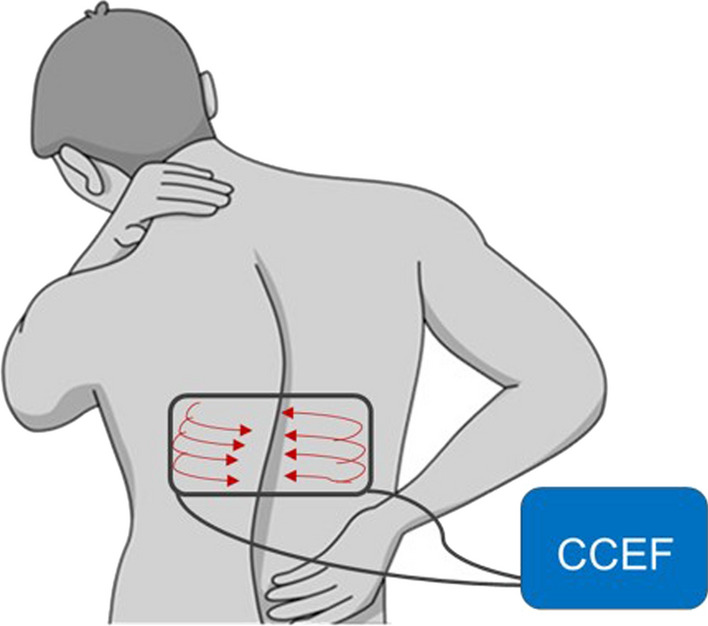
Schematic showing the correct electrode positioning during the use of the CCEF device

In the CCEF group, patients were taught and asked to place the pad paraspinal at the fracture level (Fig. 1).

### Data collection and clinical assessment

At baseline (T0), the 30-day follow-up (T1), the 60-day follow-up (T2), and the 6-month follow-up (T3), each patient underwent clinical evaluation using the Visual Analogue Scale (VAS) for Pain and the Oswestry Disability Index (ODI).

Analgesic therapy with paracetamol 1000 mg tablets for 7 days—or longer, depending on the pain intensity—was performed; patients were required to report their paracetamol consumption between study day 8 and 180 days of follow-up on a specific sheet.

The CCEF device’s internal clock enabled the patient’s compliance to be monitored according to the following formula: adherence = (recorded hours of treatment/expected hours of treatment) × 100.

Previous clinical trials of CCEF stimulation were analysed to identify the minimum adherence request. In the study by Goodwin et al. [13], patients were required to use CCEF stimulation for 24 h per day, but an average time of 15.7 h per day was recorded. Rossini et al. [11] asked the patients to use the CCEF device for at least 10 h per day, and an average time of 8.6 h per day was recorded for the stimulated group. Therefore, based on these findings, patients with an adherence of ≥ 80% were considered compliant.

To evaluate the VBME reduction independently of the patient’s compliance, the oedema reduction corrected for the hours of treatment (Δ*E*) was calculated as follows: Δ*E* = baseline oedema − oedema on the 60th day / hours of treatment.

### MRI study protocol and assessment

MRI plays a central role in diagnosing VVFs, since acute fractures show a decreased signal intensity on T1-weighted spin-echo images and a markedly increased intensity on STIR (short tau inversion recovery) T2-weighted fast spin-echo images with fat saturation [6]. This MRI pattern depends on the focal increase in water content due to microfractures within the trabecular bone and resultant haemorrhage [6].

MRI studies of the thoracolumbar spine were performed at 0 days (T0), 30 days (T1), and 60 days (T2) of follow-up using a 1.5-T MRI system at all of the centres that took part in the study.

Sagittal T1-weighted spin-echo imaging (TR, 357.0 ms; TE, 15.0 ms; slice thickness, 13.0 mm; FOV, 300 × 300 mm; matrix size, 380 × 224) and fat-suppressed T2-weighted spin-echo imaging (TR, 4500.0 ms; TE, 46.4 ms; slice thickness, 13.0 mm; FOV, 400 × 400 mm; 380 × 224) were performed along the left pedicle, the spinous process and the right pedicle.

An orthopaedic surgeon with more than 10 years of experience in spine surgery analysed the signal intensities of these three MRI slices in a blinded manner. VBME was quantified by applying the method of Piazzolla et al. [5] using DICOM software (Surgimap, version 2.3.21, Nemaris, Inc.). Two regions of interest (ROIs), the fractured vertebral body and the VBME zone, were identified manually in T2 fat-saturated sequences, taking care not to include the cortex. The areas in mm^2^ of both the ROIs and the ratio of these areas expressed as a percentage were calculated. VBME was defined as the mean of the ratios calculated for the three MRI slices.

On MRI at baseline (T0), at 30 days of follow-up (T1), and at 60 days of follow-up (T2), the vertebral body geometry was assessed by calculating the anterior wall height (AH), the posterior wall height (PH) and the ratio between them (AH/PH) as well as the vertebral kyphosis angle (VK).

### Statistical analysis

Based on previously published data [5, 6], a sample size of 16 per group achieves an 80% power to reject the null hypothesis of equal means when the population mean difference is 6.0 with a standard deviation for both groups of 5.0 and with a significance level (*a*) of 0.05 using a two-sided equal-variance *t* test.

Statistical analysis was performed using SPSS^®^ 23.0 software (SPSS Inc., Chicago, IL, USA). The Shapiro–Wilk test was conducted to verify a normal distribution of the data.

The unpaired *t* test after ANOVA and the chi-square test were used to exclude any significant difference between the two groups at baseline.

The unpaired *t* test after ANOVA was used to compare the VBME, AH/PH, VK, VAS, and ODI values between the two groups at baseline (T0), at 30 days of follow-up (T1), and at 60 days of follow-up (T2). The chi-square test was used to compare the percentage of paracetamol consumption at 30 days (T1), 60 days (T2), and 180 days (T3) of follow-up.

A paired* t* test was used to compare the VBME, AH/PH, VK, VAS and ODI values at each follow-up versus baseline within the same group. The tests were two-tailed. A *p* value of less than 0.05 was considered significant.

## Results

A total of 71 patients were included in the present study. Three patients out of 71 (4.23%) were excluded because they refused to perform a closed MRI examination, whereas 2 patients out of 68 (2.94%) were lost to follow-up.

A total of 66 patients (male: 9, 13.63%; female: 57, 86.37%; mean age: 73.15 years old) with 69 VFFs were included in the present study and randomized as follows: 33 patients were included in the control group and the remaining 33 patients were randomized into the CCEF group.

At baseline, no significant differences between groups were observed in terms of sex percentage, mean age, BMI, smoking status and vertebral fracture site (Table 1).

**Table 1:** Main data from the study

	Control group	CCEF group	*p* value
Patients, *n* (VFFs)	33 (VFFs = 34)	33 (VFFs = 35)	–
Gender			
Male, *n* (%)	4 (12.12%)	5 (15.15%)	0.65
Age			
Mean ± SD	72.88 ± 6.09	73.6 ± 7.82	0.768
Range	65–84	64–83	–
BMI (kg/m^2^)			
Mean ± SD	26.055 ± 3.9	26.735 ± 4.35	0.722
Smoking status			
*N* of smokers (%)	10 (30.3%)	10 (30.3%)	–
Vertebral fracture type (OF classification)			
OF 1, *n* (%)	1 (2.94%)	2 (5.7%)	0.03*
OF 2, *n* (%)	33 (97.06%)	33 (94.3%)	0.07
Vertebral fracture site			
T10, *n* (%)	3 (8.82%)	3 (8.57%)	0.83
T11, *n* (%)	4 (11.76%)	3 (8.57%)	0.83
T12, *n* (%)	9 (26.47%)	10 (28.6%)	0.21
L1, *n* (%)	10 (29.42%)	11 (31.4%)	0.11
L2, *n* (%)	5 (14.71%)	5 (14.29%)	0.88
L3, *n* (%)	3 (8.82%)	3 (8.57%)	0.83
Adverse reactions to CCEF			
*N* (%)	0	N/A	–
Patient compliance			
Adherence (%)	94%	N/A	–
Oedema resolution per hour of CCEF therapy (mm^2^/h)			
Δ*E* (mean ± SD)	0.95 ± 0.47	N/A	–

*N/A* not available

^*^p < 0.005 (chi-square test)

In the CCEF group, very high patient compliance (adherence = 94%) was observed in the absence of CCEF-therapy-related adverse effects (Table 1). The main data from the study are summarized in Table 1.

### MRI outcome

**Fig. 2:**
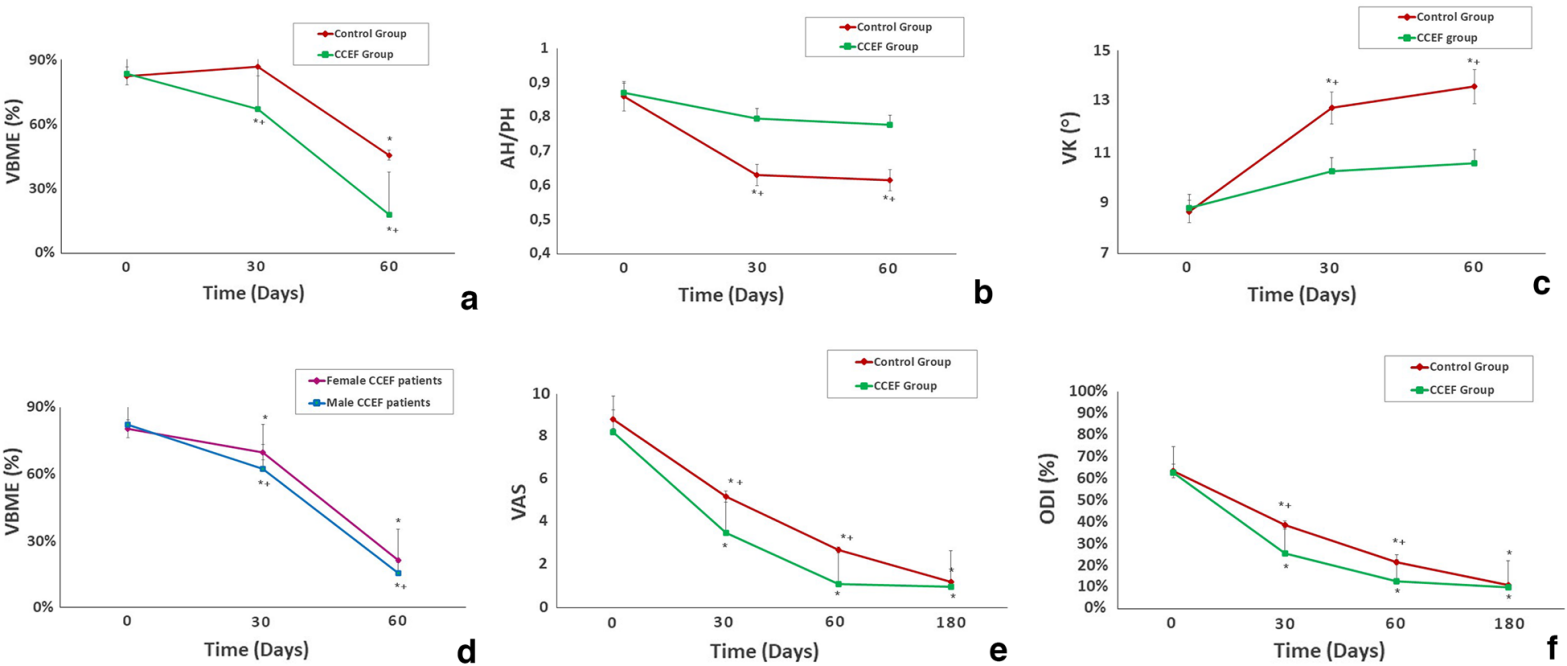
Summary of MRI and clinical data. **A** The mean VBME values in both groups at baseline and follow-ups. **B** The mean AH/PH ratio values in both groups at baseline and follow-ups. **C** The mean VK values in both groups at baseline and follow-ups. **D** The mean VBME values in male versus female patients of the CCEF group at baseline and follow-ups. **E** The mean VAS values in both groups at baseline and follow-ups. **F** The mean ODI values in both groups at baseline and follow-ups. The* error bars* indicate the standard deviation. *p < 0.05 (paired* t* test); ^+^*p* < 0.05 (unpaired* t* test)

Faster VBME resolution was observed in the CCEF group at the 30-day follow-up (*p* = 0.002) and the 60-day follow-up (*p* < 0.001) (Tables 1, 2; Fig. 2A). Overall, a Δ*E* of 0.95 ± 0.47 mm^2^ for each hour of CCEF stimulation performed was observed in the CCEF group (Tables 1, 2).

**Table 2:** Comparison between the two groups: VBME, AH/PH ratio, VK (unpaired *t* test)

	VBME			AH/PH			VK		
	Control group	CCEF group	*p* value	Control group	CCEF group	*p* value	Control group	CCEF group	*p* value
Baseline (mean ± SD)	0.823 ± 0.06	0.835 ± 0.07	0.843	0.86 ± 0.06	0.87 ± 0.04	0.745	8.65 ± 4.44	8.79 ± 5.67	0.895
30-day follow-up (mean ± SD)	0.866 ± 0.06	0.67 ± 0.06	0.002*	0.63 ± 0.11	0.805 ± 0.09	0.02*	13.73 ± 5.67	10.24 ± 4.48	0.031*
60-day follow-up (mean ± SD)	0.455 ± 0.08	0.18 ± 0.11	< 0.001*	0.615 ± 0.13	0.786 ± 0.12	0.001*	14.57 ± 5.06	10.55 ± 5.16	0.046*

*VBME* vertebral bone marrow oedema, *AH/PH* anterior wall height/posterior wall height ratio,* VK* vertebral kyphosis

*p < 0.05 (unpaired *t* test)

The AH/PH ratio and VK angle were impaired in both groups at the 30-day and 60-day follow-ups (Tables 2, 3; Fig. 2B, C), but a significantly higher AH/PH ratio and level of VK impairment was observed in the control group at the 30-day follow-up (*p* = 0.02 and *p* = 0.031, respectively) and 60-day follow-up (*p* = 0.001 and *p* = 0.046, respectively).

**Table 3:** Comparison of the VBME, AH/PH ratio and VK differences from baseline at each follow-up within the same group (paired* t* test)

	Control group			CCEF group		
	VBME	AH/PH	VK	VBME	AH/PH	VK
30 days of follow-up	0.647	0.002*	0.02*	0.01*	0.075	0.0575
60 days of follow-up	0.003*	0.001*	0.008*	0.001*	0.06	0.087

*VBME* vertebral bone marrow oedema, *AH/PH* anterior wall height/posterior wall height ratio,* VK* vertebral kyphosis

*p < 0.005 (paired* t* test)

Hence, at the 60-day follow-up, significantly better vertebral body shape preservation was observed in the CCEF group (Fig. 2C and D).

Furthermore, a sex-related difference in VBME resolution time was observed in the CCEF group: although both sexes were not equally represented in this study, since osteoporosis presents a higher incidence in females, a significantly faster VBME resolution was observed in males compared to females (Fig. 2D).

**Fig. 3:**
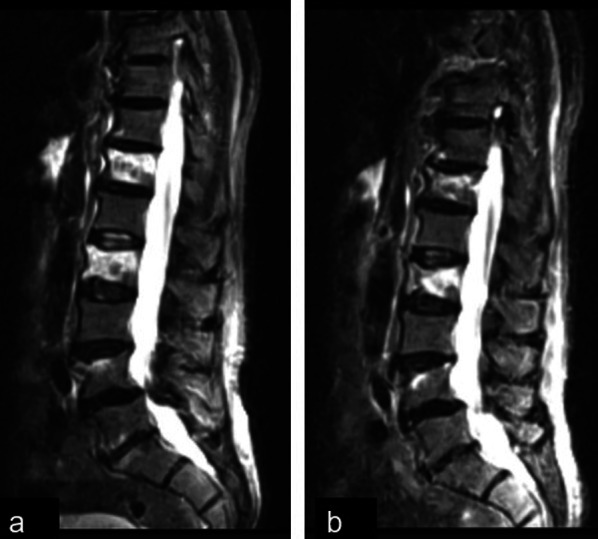
Baseline MRI (**a**) and 60-day follow-up MRI (**b**) of a female patient belonging to the CCEF group. All the images show a STIR sequence

**Fig. 4:**
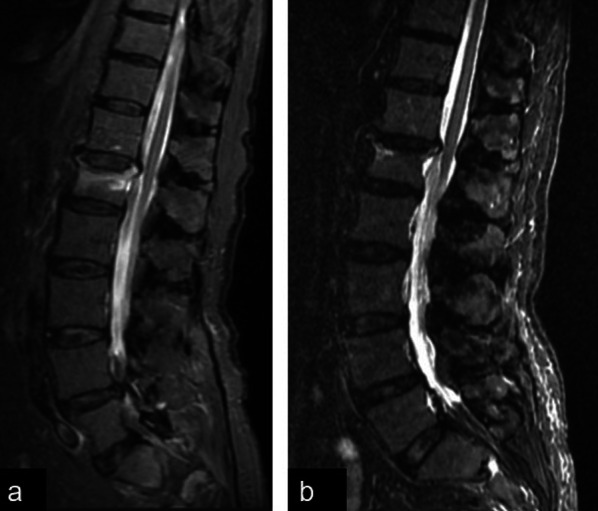
Baseline MRI (**a**) and 60-day follow-up MRI (**b**) of a female patient belonging to the CCEF group. All the images show a STIR sequence

Figures 3 and 4 show the MRIs at baseline and at the 6-month follow-up of a Control patient (Fig. 3) and a Stimulated patient (Fig. 4).

### Clinical outcome

In the CCEF group, the quicker VBME resolution had a positive impact on the pain and quality of life improvement. Therefore, a faster mean VAS reduction and ODI mean score improvement were observed in the CCEF group (Tables 4, 5; Fig. 2E, F).

**Table 4:** Comparison between the two groups: VAS, ODI and paracetamol consumption (unpaired *t* test)

	VAS			ODI			Paracetamol consumption		
	Control group	CCEF group	*p* value	Control group	CCEF group	*p* value	Control group	CCEF group	*p* value
Baseline (mean ± SD)	8.8 ± 0.7	8.2 ± 1.2	0.644	0.63 ± 0.04	0.625 ± 0.06	0.644	–	–	–
30-day follow-up (mean ± SD)	5.2 ± 1.3	3.5 ± 0.8	0.01*	0.38 ± 0.05	0.254 ± 0.07	0.01*	0.86 ± 0.16	0.723 ± 0.14	0.033*
60-day follow-up (mean ± SD)	2.7 ± 0.4	1.1 ± 0.3	0.02*	0.21 ± 0.04	0.127 ± 0.03	0.01*	0.544 ± 0.25	0.224 ± 0.18	0.001*
180-day follow-up (mean ± SD)	1.02 ± 0.79	0.98 ± 0.0.88	0.544	0.11 ± 0.75	0.10 ± 0.97	0.424	0.17 ± 0.11	0.09 ± 0.07	0.001*

*p < 0.005 (unpaired *t* test)

**Table 5:** Comparison of VAS and ODI within the same group at each follow-up versus baseline (paired *t* test)

	Control group		CCEF group	
	VAS	ODI	VAS	ODI
30 days of follow-up	0.003*	0.002*	0.001	0.001*
60 days of follow-up	0.001*	0.001*	0.001	< 0.001*
180 days of follow-up	< 0.001*	< 0.001*	< 0.001	< 0.001*

*p < 0.005 (paired *t* test)

Moreover, a significantly lower paracetamol consumption rate was observed from the third follow-up after treatment until the 6-month follow-up in the CCEF group.

**Fig. 5:**
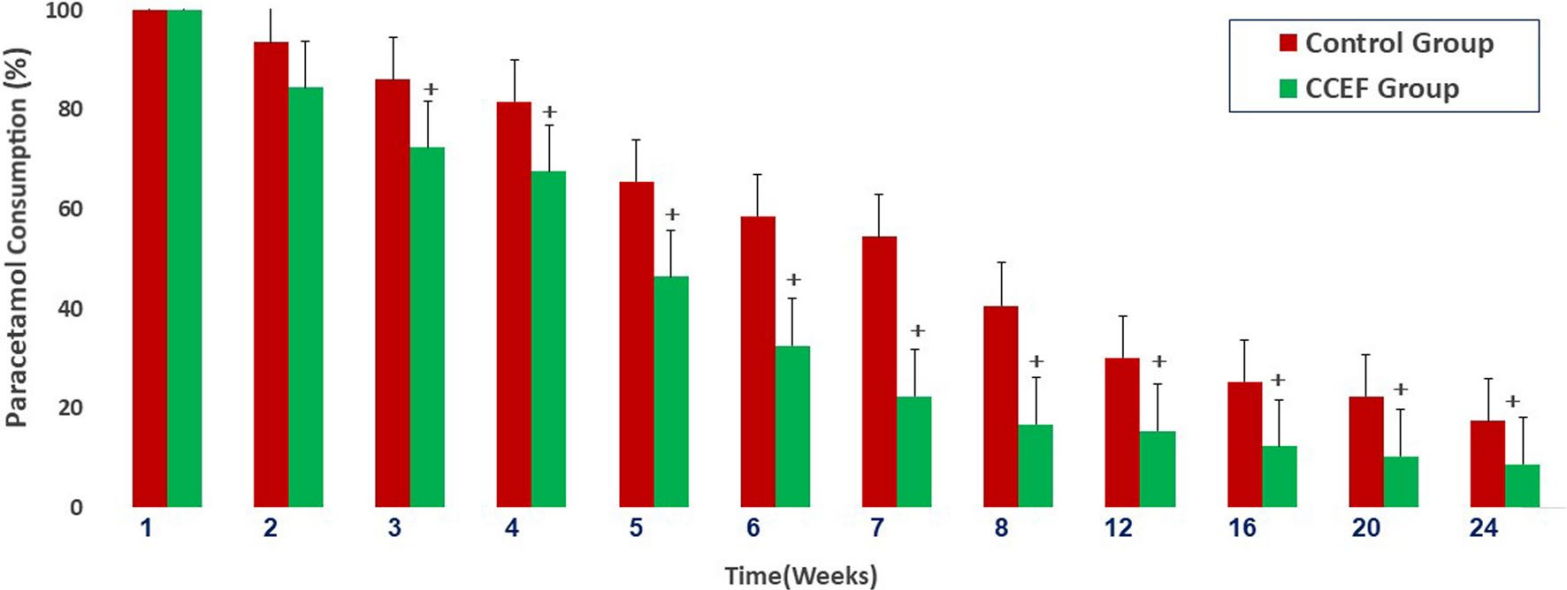
Paracetamol consumption in both groups at baseline and follow-ups. The* error bars* indicate the standard deviation. ^+^*p* < 0.05 (unpaired* t* test)

The faster VBME resolution, together with the prevention of significant vertebral body collapse, had a positive impact on the stimulated patients’ clinical symptoms and reduced the paracetamol consumption up to the 6-month follow-up. A significantly lower paracetamol consumption has been observed in the stimulated group three weeks after recruitment (Fig. 5).

### Adverse effects

Up to the 6-month follow-up, no adverse effects were observed in either the study group or the control group. Biophysical stimulation with CCEFs was revealed to be clinically safe and effective (according to MRI) for the treatment of VFFs.

## Discussion

VBME in MRI is the gold standard for the diagnosis of acute VFFs. Recent VFFs are hypointense in T1 and hyperintense in STIR sequences, whereas chronic VFFs appear isointense in all MRI sequences [6, 14]. Consequently, VBME can be monitored to assess the VFF healing process, thus showing the effectiveness of a conservative therapeutic protocol for a vertebral fracture.

In the present study, we have hypothesized that CCEF, in combination with the standard conservative protocol for the treatment of VFFs, could enhance fracture healing by hastening VBME resolution and improving clinical symptoms.

CCEF is a non-invasive bone growth stimulator used in clinical practice to enhance endogenous osteogenesis. In vitro studies on MC3T3-E1 bone cells have explained the mechanism of action for CCEF, which involves opening plasma membrane voltage-gated calcium channels followed by an increased cytosolic calcium concentration and subsequent phospholipase A2 (PLA2) activity [15]. The cytosolic calcium increase activates the calmodulin pathway, thus resulting in upregulated expression of osteogenic genes, including fibroblast growth factor (FGF)-2, osteocalcin (BGP), transforming growth factor-β superfamily genes (i.e. TGF-β1, -β2 and -β3; BMP-2 and -4) and alkaline phosphatase (ALP) [16, 17]. PLA2 also acts by increasing the synthesis of prostaglandin E2 (PGE2), which promotes osteogenesis by raising the cellular L-ascorbic acid uptake through the membrane carrier sodium vitamin C transporter-2 (SVCT-2) [18].

The positive effects of CCEF stimulation on fracture healing in animal models have been reported by different authors. CCEF in a castration-induced osteoporosis animal modelwas able to restore the bone mass/unit volume in the rat vertebral body [19]. Gan et al. [20], using a rabbit posterolateral intertransverse spinal fusion model, found that there was a greater percentage of bilateral and unilateral fusion in a CCEF-stimulated group compared to a control group.

CCEF stimulation is currently used in the treatment of recalcitrant non-union healing [21, 22], as an adjunct in the treatment of vertebral fusions [12, 13], in the treatment of acute VFFs [5] and in patients with chronic VFFs to reduce NSAID consumption for chronic pain [11].

The present study is the first multicentre RCT aiming to assess the effectiveness of CCEF at resolving VBME and back pain and improving quality of life in patients suffering from VFFs at the 6-month follow-up. Hence, the healing times of 34 VFFs in 33 patients who were conservatively treated with bed rest for 20 days, subsequently mobilized with a brace, and given vitamin D supplementation and antiresorptive therapy were compared to the evolution of 35 VFFs in 33 patients who received CCEF stimulation as an adjunct. The two groups were comparable in terms of sex representation, mean age, BMI, smoking status and vertebral fracture site.

In the CCEF group, good compliance was observed in CCEF therapy (adherence = 94%), and no adverse effects were recorded.

The most important finding of the present study was that CCEF therapy hastens VBME resolution in VFFs and prevents vertebral body deformation; this more rapid fracture healing has a positive impact on back pain (VAS), quality of life (ODI) and paracetamol consumption compared to non-stimulated patients.

Hence, in the stimulated patients, we detected faster VBME resolution: the patients’ mean VBME in the CCEF group was significantly lower than that in the control group at both the 30-day and 60-day follow-ups (Table 2; Fig. 2A).

The action of capacitive biophysical stimulation on VBME resolution is highly supported by preclinical evidence in vitro and in vivo that explained the mechanisms of action of CCEF and has been further validated in clinical trials [15–20].

Interestingly, the VBME resolved more quickly in male CCEF patients compared to females. This sex-related difference, which is probably due to gender differences in the expression and activation of TGF-β superfamily genes in skeletal muscle and bone [23, 24], could lead to the development of a gender-specific biophysical stimulation protocol in the management of VFFs.

On the other hand, in the control group, a non-significant VBME percentage increase was observed at 30 days follow-up (Table 3; Fig. 2A), depending on significant vertebral body collapse (Tables 2, 3; Fig. 2B, C). It is noteworthy that the AH/PH ratio was impaired in both groups at the 30-day follow-up but that significantly higher AH/PH impairment was observed in the control group compared with the CCEF group (Table 2; Fig. 2B). Mean VK also showed comparable trends in both groups during follow-up (Table 2; Fig. 2C). These findings highlight that CCEF therapy, together with bed rest followed by bracing, is effective in preventing vertebral body collapse in acute VFFs.

It is also remarkable that a sex-related difference in VBME resolution was observed in the stimulated patients: stimulated male patients showed significantly faster VBME resolution compared to females; thus, CCEF was revealed to be more effective in male patients in the present study.

In the CCEF group, faster pain reduction (mean reduction in VAS) and an ODI mean score improvement were observed (Tables 4, 5; Fig. 2E, F). Therefore, the faster VBME resolution, together with the prevention of significant vertebral body collapse, had a positive impact on the stimulated patients’ clinical symptoms.

In the CCEF group, a significantly lower paracetamol consumption rate was observed from the third follow-up after treatment until the 6-month follow-up. CCEF therapy, as already shown in a previous study by Rossini et al. [11], also positively impacts analgesic drug discontinuation in patients recovering from VFFs.

Rossini et al. [11], nonetheless, investigated the role of CCEF therapy in patients with multiple chronic VFFs and concomitant chronic back pain, documenting a higher percentage of non-steroidal anti-inflammatory drug (NSAID) discontinuation in stimulated patients. Those authors did not investigate the role of CCEF therapy in patients with acute VFFs, so our study complements the investigation conducted by Rossini et al. by documenting the effectiveness of CCEF in the treatment of acute VFFs.

Furthermore, our findings confirm that, as observed in a preliminary study by Piazzolla et al. [5], CCEF used as an adjunct to traditional conservative treatment may hasten the fracture healing process, thus reducing the patient’s back pain and analgesic drug consumption and enhancing the patient’s recovery. In a preliminary study by Piazzolla et al. [5], quicker VBME resolution was observed compared to the present study, but the significance of the MRI and clinical data is comparable in both studies. The difference between the two studies’ findings may be explained by considering the different mean ages of the patients recruited in the two studies: 63.8 years old in the preliminary study [5] and 73.15 years old in the current study.

Based on the present study findings, CCEF is effective in improving acute VFF healing, thus preventing prolonged patient immobilization and bed-related complications. Hence, the use of this non-invasive biophysical stimulation as an adjunct to the traditional conservative protocol should be highly encouraged in daily clinical practice to hasten the recovery time from non-operatively managed acute VFFs.

Physicians should consider these data in daily clinical practice decision making, and policymakers should consider introducing capacitive biophysical stimulation, as an adjunct to conservative management, in the guidelines for the management of acute VFFs.

The main limitation of the present study is the lack of a placebo device in the control group. However, the effectiveness of capacitive biophysical stimulation over a placebo device has already been documented by Rossini et al. [11] and Massari et al. [12]; consequently, the use of a placebo device was considered unnecessary in this study. Furthermore, all the study data were gathered and analysed blindly.

Future studies are needed to assess whether capacitive biophysical stimulation could be useful in preventing acute VFFs in osteoporotic patients.

Moreover, considering the crosstalk between bone and muscle and the concomitant sarcopenia in osteoporotic patients, future studies should investigate the role of CCEF in the treatment and prevention of paraspinal sarcopenia in patients suffering from osteosarcopenia.

## Conclusions

Biophysical stimulation with CCEF, as an adjunct to traditional conservative treatment, hastens VBME resolution and prevents vertebral body deformation in acute VFFs. These radiologic findings correlate with faster back pain resolution (VAS), quicker quality of life improvement (ODI) and reduced paracetamol consumption.

## Data Availability

The data that support the findings of this study are available on request from the corresponding author.
